# Supplementary material for: Integrated Fluidic Platform for Washing and Mechanical Processing of Lipoaspirate for Downstream Fat Grafting and Regenerative Applications
Source: Bioengineering (Basel). 2025 Aug 26;12(9):918. doi: 10.3390/bioengineering12090918 (PMC12467523; doi:10.3390/bioengineering12090918)
Supplement: Supplementary file 1 [file bioengineering-12-00918-s001.zip › bioengineering-3818024-supplementary.pdf]

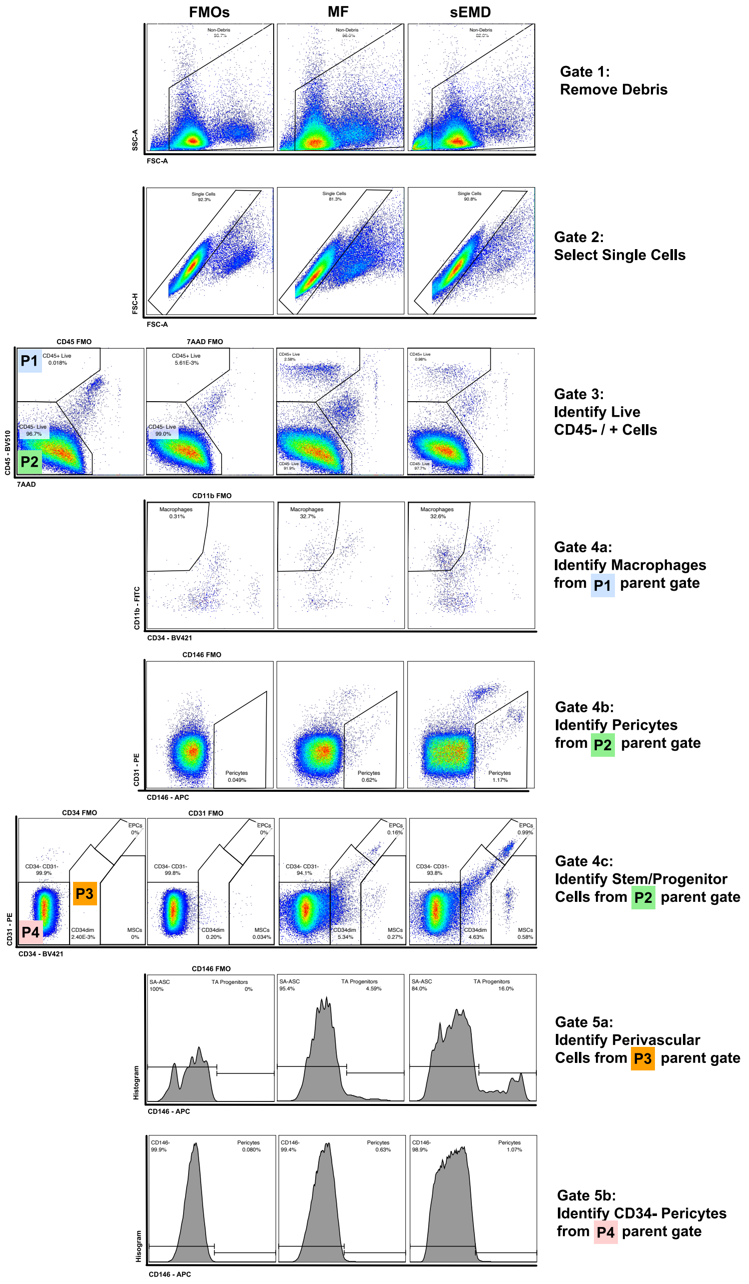

Figure S1. Flow cytometry gating scheme. SVF cell suspensions obtained after collagenase I digestions were stained with fluorescent probes (listed in Table 1) and signals were assessed by flow cytometry. Data was then analyzed on FlowJo using a sequential gating scheme for stem and progenitor cell types of interest (listed in Table 2). Gate 1 was used to exclude debris near the origin using FSC-A vs SSC-A. Gate 2 used FSC-A vs FSC-H to select single cells. Gate 3 used 7AAD vs CD45-BV510 to distinguish live CD45+ and CD45- populations. Gate 4a was gated as a subset of the P1 population with CD34-BV421 vs CD11b-FITC to distinguish macrophages. Gate 4b was gated as a subset of the P2 population with CD146-APC vs CD31-PE to distinguish pericytes. Gate 4c was gated as a subset of the P2 population with CD34-BV421 vs CD31-PE to distinguish CD34^{dim} cells, MSCs, and EPCs. Gate 5a was gated as a subset of the P3 population with CD146-APC vs histogram to distinguish CD34^{dim} SA-ASC and CD34^{dim} TA Progenitor cells. Gate 5b was gated as a subset of the P4 population with CD146-APC vs histogram to distinguish CD34- pericytes.

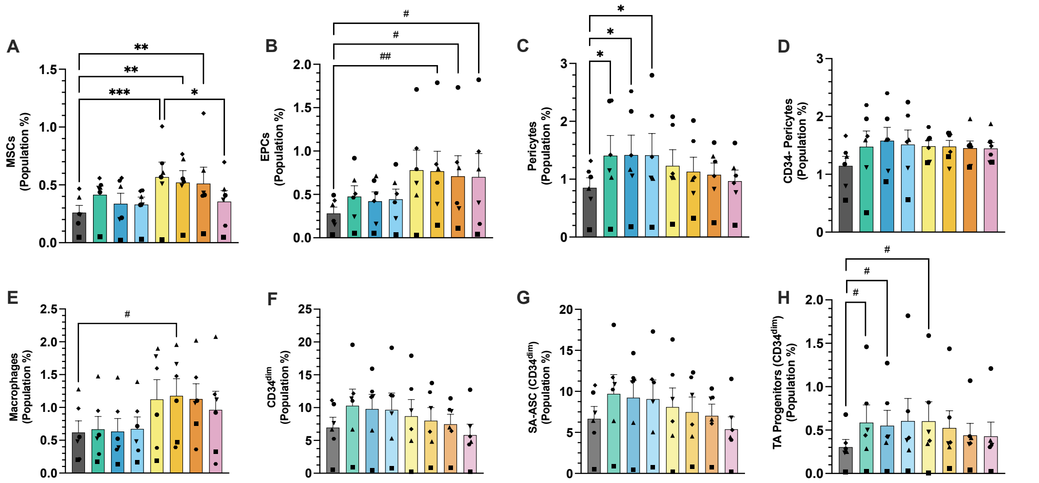

Figure S2. Population percentages for normalized flow cytometry data in Figure 4. Each graph shows the mean ± SEM, with individual data points representing independent donor samples (n=6 donors per condition). Values are expressed as a percentage of the total population obtained via flow cytometry. Error bars represent standard error from at least 3 independent experiments. (**A**) MSCs, (**B**) EPCs, (**C**) pericytes, (**D**) CD34- pericytes, (**E**) macrophages, (**F**) CD34^{dim} cells, (**G**) SA-ASC, and (**H**) TA progenitors. Graphs that violated assumptions for two-way ANOVA were analyzed using a non-parametric Friedman test with Dunn's post hoc comparisons. Holm–Šidák post hoc; *$p < 0.05$, **$p < 0.01$, ***$p < 0.001$. Dunn post hoc: #$p < 0.05$, ##$p < 0.01$.

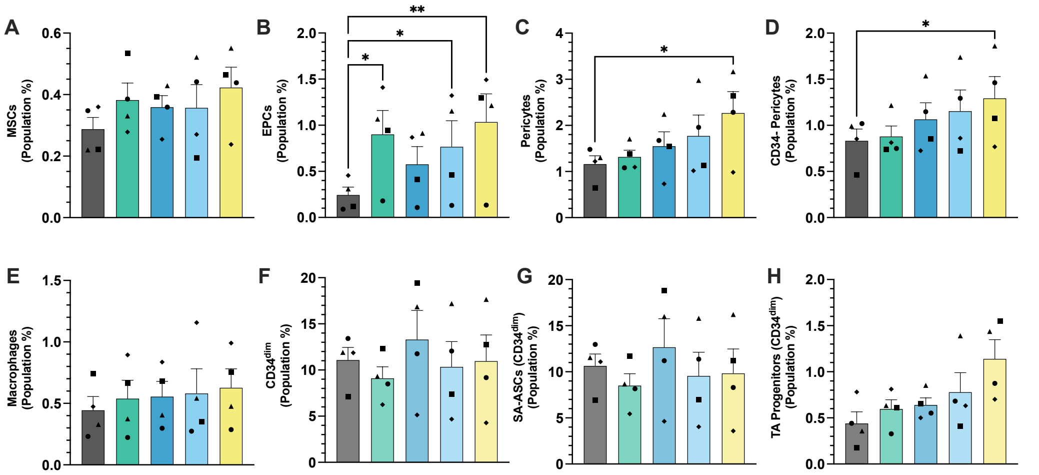

Figure S3. Population percentages for normalized flow cytometry data in Figure 5. Each graph shows the mean ± SEM, with individual data points representing independent donor samples (n=4 donors per condition). Values are expressed as a percentage of the total population obtained via flow cytometry. Error bars represent standard error from at least 3 independent experiments. (**A**) MSCs, (**B**) EPCs, (**C**) pericytes, (**D**) CD34- pericytes, (**E**) macrophages, (**F**) CD34^{dim} cells, (**G**) SA-ASC, and (**H**) TA progenitors. Graphs that violated assumptions for two-way ANOVA were analyzed using a non-parametric Friedman test with Dunn's post hoc comparisons. Holm–Šidák post hoc; *$p < 0.05$, **$p < 0.01$.

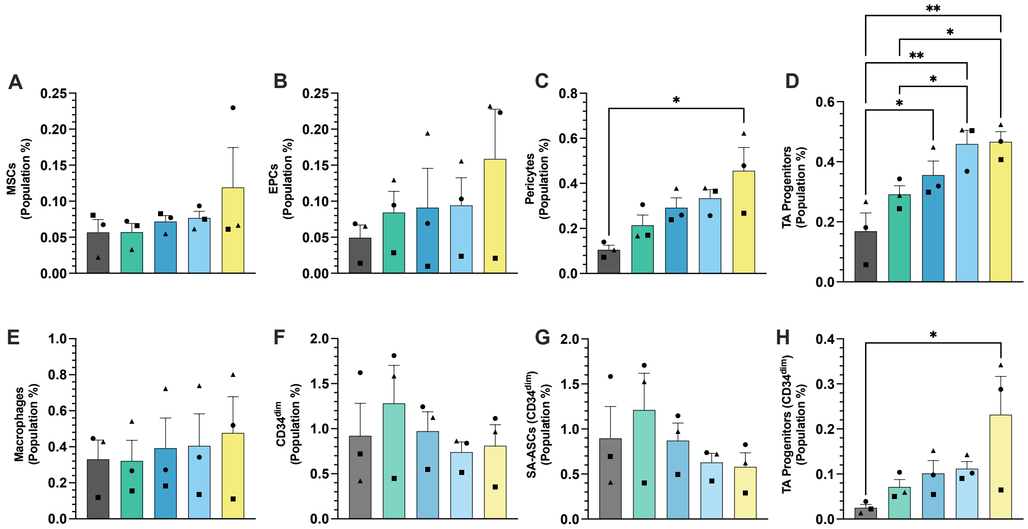

Figure S4. Population percentages for normalized flow cytometry data in Figure 6. Each graph shows the mean ± SEM, with individual data points representing independent donor samples (n=3 donors per condition). Values are expressed as a percentage of the total population obtained via flow cytometry. Error bars represent standard error from at least 3 independent experiments. (**A**) MSCs, (**B**) EPCs, (**C**) pericytes, (**D**) CD34- pericytes, (**E**) macrophages, (**F**) CD34^{dim} cells, (**G**) SA-ASC, and (**H**) TA progenitors. Graphs that violated assumptions for two-way ANOVA were analyzed using a non-parametric Friedman test with Dunn's post hoc comparisons. Holm–Šidák post hoc; *$p < 0.05$, **$p < 0.01$.

Table S1. Summary of cell counts and population percentages (Mean ± SD) in Figure 4.

	Mean ± SD							
**Outcomes**	**MF**	**1 min**	**3 min**	**5 min**	**sEMD**	**1 min + sEMD**	**3 min + sEMD**	**5 min + sEMD**
Count	627,167 ±293,798	769,417 ±305,629	685,500 ±294,780	635,417 ±228,213	545,083 ±230,843	565,167 ±198,681	542,167 ±156,007	649,167 ±160,917
Viability	88 ± 4.8	88 ± 5.7	90 ± 4.0	89 ± 2.9	89 ± 2.6	87 ± 3.1	86 ± 3.1	88 ± 2.5
**Population (%, normalized to MF)**								
MSCs	–	1.7 ± 0.60	1.2 ± 0.56	1.3 ± 0.51	2.1 ± 0.97	2.1 ± 0.66	1.9 ± 0.40	1.4 ± 0.63
EPCs	–	1.7 ± 0.40	1.5 ± 0.61	1.5 ± 0.39	2.7 ± 1.5	2.9 ± 0.82	2.5 ± 0.61	2.1 ± 1.1
Pericytes	–	1.6 ± 0.54	1.6 ± 0.45	1.6 ± 0.60	1.5 ± 0.42	1.5 ± 0.62	1.4 ± 0.39	1.2 ± 0.35
CD34^{-} Pericytes	–	1.2 ± 0.40	1.4 ± 0.30	1.3 ± 0.36	1.4 ± 0.43	1.4 ± 0.51	1.4 ± 0.39	1.4 ± 0.47
Macrophages	–	1.1 ± 0.17	1.0 ± 0.35	1.1 ± 0.28	1.9 ± 0.95	2.1 ± 0.73	2.1 ± 0.82	1.5 ± 0.56
CD34^{dim}	–	1.5 ± 0.43	1.4 ± 0.56	1.4 ± 0.53	1.2 ± 0.78	1.3 ± 0.64	1.2 ± 0.42	0.77 ± 0.30
SA-ASC (CD34^{dim})	–	1.5 ± 0.42	1.4 ± 0.56	1.4 ± 0.52	1.2 ± 0.78	1.3 ± 0.64	1.2 ± 0.44	0.74 ± 0.29
TA Progenitors (CD34^{dim})	–	1.8 ± 0.65	1.8 ± 0.58	1.8 ± 0.67	1.7 ± 0.88	1.9 ± 0.92	1.6 ± 0.45	1.4 ± 0.32

Table S2. Summary of statistical analyses in Figure 4.

Outcomes	Test Used	Factors Tested	Test Statistic (df)	Main Effect p-values	Post Hoc Test	Significant Comparisons	Notes/Assumptions
Count	Friedman	Condition (Repeated)	$\chi^2(7) = 5.444$	$p = 0.6059$	–	–	Failed Spearman's test (p = 0.0405), normality passed
Viability	Two-way ANOVA	Patient, Condition	F(5,35) = 2.843 (Patient), F(7,35) = 0.7624 (Condition)	Patient: 0.0295, Condition: 0.6221	–	–	Assumptions met
MSCs	Two-way ANOVA	Patient, Condition	F(5,35) = 27.88 (Patient), F(7,35) = 5.405 (Condition)	Patient: <0.0001, Condition: 0.0003	Holm–Šidák	A vs. E (p = 0.0005), A vs. F (p = 0.0037), A vs. G (p = 0.0049), E vs. H (p = 0.0218)	Assumptions met
EPCs	Friedman	Condition (Repeated)	$\chi^2(7) = 19.56$	$p = 0.0066$	Dunn	A vs. F (p = 0.0026), A vs. G (p = 0.0218), A vs. H (p = 0.0468)	Failed Spearman's (p = 0.0063), normality passed
Pericytes	Two-way ANOVA	Patient, Condition	F(5,35) = 38.51 (Patient), F(7,35) = 3.439 (Condition)	Patient: <0.0001, Condition: 0.0066	Holm–Šidák	A vs. B/C/D (p = 0.0164)	Assumptions met
CD34^{-} Pericytes	Two-way ANOVA	Patient, Condition	F(5,35) = 14.68 (Patient), F(7,35) = 1.370 (Condition)	Patient: <0.0001, Condition: 0.2485	–	–	Assumptions met
Macrophages	Friedman	Condition (Repeated)	$\chi^2(7) = 28.17$	$p = 0.0002$	Dunn	A vs. F (p = 0.0322)	Failed Spearman's (p = 0.0379), Failed D'Agostino–Pearson (p = 0.0367)
CD34^{dim}	Two-way ANOVA	Patient, Condition	F(5,35) = 44.15 (Patient), F(7,35) = 3.446 (Condition)	Patient: <0.0001, Condition: 0.0066	Holm–Šidák	None significant	Assumptions met
SA-ASC (CD34^{dim})	Two-way ANOVA	Patient, Condition	F(5,35) = 41.57 (Patient), F(7,35) = 3.447 (Condition)	Patient: <0.0001, Condition: 0.0066	Holm–Šidák	None significant	Assumptions met
TA Progenitors (CD34^{dim})	Friedman	Condition (Repeated)	$\chi^2(7) = 16.78$	$p = 0.0189$	Dunn	A vs. B (p = 0.0146), A vs. C (p = 0.0322), A vs. E (p = 0.0468)	Passed Spearman's (p = 0.1338), Failed D'Agostino–Pearson (p = 0.0377)

Comparison Key: MF [A], 1 min [B], 3 min [C], 5 min [D], sEMD [E], 1 min + sEMD [F], 3 min + sEMD [G], 5 min + sEMD [H]

Table S3. Summary of cell counts and population percentages (Mean ± SD) in Figure 5.

**Outcomes**	**Mean ± SD**				
	**MF**	**sEMD**	**pEMD (30 sec)**	**pEMD (60 sec)**	**pEMD (90 sec)**
Count	702,000 ±343,738	614,375 ±347,441	663,000 ±163,205	621,625 ±229,700	628,750 ±240,568
Viability	88 ± 6.9	82 ± 3.3	82 ± 5.7	82 ± 5.0	81 ± 4.2
**Population (%, normalized to MF)**					
MSCs	–	1.4 ± 0.71	1.4 ± 0.59	1.3 ± 0.74	1.6 ± 0.83
EPCs	–	4.1 ± 2.6	2.4 ± 1.0	3.0 ± 1.1	4.9 ± 4.1
Pericytes	–	1.3 ± 0.63	1.5 ± 0.78	1.6 ± 0.62	2.2 ± 1.4
$CD34^-$ Pericytes	–	1.1 ± 0.37	1.3 ± 0.44	1.4 ± 0.32	1.6 ± 0.61
Macrophages	–	1.2 ± 0.46	1.3 ± 0.35	1.4 ± 0.83	1.4 ± 0.46
$CD34^{dim}$	–	0.92 ± 0.55	1.4 ± 1.0	0.94 ± 0.43	1.1 ± 0.67
SA-ASC ($CD34^{dim}$)	–	0.89 ± 0.54	1.3 ± 0.99	0.90 ± 0.42	1.0 ± 0.61
TA Progenitors ($CD34^{dim}$)	–	1.8 ± 1.2	2.0 ± 1.4	2.1 ± 1.3	3.9 ± 3.5

Table S4. Summary of statistical analyses in Figure 5.

Outcomes	Test Used	Factors Tested	Test Statistic (df)	Main Effect p-values	Post Hoc Test	Significant Comparisons	Notes/Assumptions
Count	Friedman	Condition (Repeated)	$\chi^2(4) = 3.291$	$p = 0.5541$	–	–	Failed Spearman's test (p = 0.0098), normality passed
Viability	Two-way ANOVA	Patient, Condition	F(3,12) = 22.00 (Patient), F(4,12) = 5.311 (Condition)	Patient: <0.0001, Condition: 0.0107	Holm–Šidák	A vs. B (p = 0.0223), A vs. C (p = 0.0194), A vs. D (p = 0.0194), A vs. E (p = 0.0157)	Assumptions met
MSCs	Two-way ANOVA	Patient, Condition	F(3,12) = 1.420 (Patient), F(4,12) = 0.8206 (Condition)	Patient: 0.2851, Condition: 0.5365	–	–	Assumptions met
EPCs	Two-way ANOVA	Patient, Condition	F(3,12) = 16.50 (Patient), F(4,12) = 6.839 (Condition)	Patient: 0.001, Condition: 0.0042	Holm–Šidák	A vs. B (p = 0.0118), A vs. D (p = 0.0422), A vs. E (p = 0.0033)	Assumptions met
Pericytes	Two-way ANOVA	Patient, Condition	F(3,12) = 6.165 (Patient), F(4,12) = 3.331 (Condition)	Patient: 0.0089, Condition: 0.0471	Holm–Šidák	A vs. E (p = 0.0438)	Assumptions met
$CD34^-$ Pericytes	Two-way ANOVA	Patient, Condition	F(3,12) = 14.45 (Patient), F(4,12) = 3.449 (Condition)	Patient: 0.0003, Condition: 0.0286	Holm–Šidák	A vs. E (p = 0.0376)	Assumptions met
Macrophages	Friedman	Condition (Repeated)	$\chi^2(4) = 6.20$	$p = 0.1972$	–	–	Failed Spearman's (p = 0.0268), Failed D'Agostino–Pearson (p = 0.0473)
$CD34^{dim}$	Two-way ANOVA	Patient, Condition	F(3,12) = 3.899 (Patient), F(4,12) = 0.6377 (Condition)	Patient: 0.0371, Condition: 0.6455	–	–	Assumptions met
SA-ASC ($CD34^{dim}$)	Two-way ANOVA	Patient, Condition	F(3,12) = 1.273 (Patient), F(4,12) = 0.0585 (Condition)	Patient: 0.3279, Condition: 0.0585	–	–	Assumptions met
TA Progenitors ($CD34^{dim}$)	Two-way ANOVA	Patient, Condition	F(3,12) = 4.178 (Patient), F(4,12) = 0.7445 (Condition)	Patient: 0.0306, Condition: 0.5800	–	–	Assumptions met

Comparison Key: MF [A], sEMD [B], pEMD (30 sec) [C], pEMD (60 sec) [D], pEMD (90 sec) [E]

Table S5. Summary of cell counts and population percentages (Mean ± SD) in Figure 6.

Outcomes	Mean ± SD				
	**MF**	**sEMD**	**iEMD (180 sec)**	**iEMD (270 sec)**	**iEMD (360 sec)**
Count	396,250 ±177,245	376,167 ±126,485	380,333 ±59,851	274,583 ±29,543	307,583 ±185,930
Viability	84 ± 6.2	90 ± 3.3	91 ± 1.0	91 ± 1.7	90 ± 2.7
**Population (%, normalized to MF)**					
MSCs	–	1.1 ± 0.34	1.5 ± 0.81	1.7 ± 0.97	2.4 ± 1.4
EPCs	–	1.8 ± 0.37	1.6 ± 1.2	1.9 ± 0.47	2.8 ± 1.1
Pericytes	–	2.1 ± 0.41	2.9 ± 0.95	3.5 ± 1.6	4.4 ± 1.4
$CD34^-$ Pericytes	–	2.4 ± 1.6	2.9 ± 2.0	4.2 ± 3.9	3.9 ± 2.8
Macrophages	–	1.1 ± 0.40	1.3 ± 0.59	1.2 ± 0.48	1.3 ± 0.49
$CD34^{dim}$	–	1.8 ± 1.7	1.4 ± 1.1	1.1 ± 0.83	1.2 ± 0.99
SA-ASC ($CD34^{dim}$)	–	1.8 ± 1.7	1.3 ± 0.96	0.95 ± 0.71	0.82 ± 0.61
TA Progenitors ($CD34^{dim}$)	–	3.1 ± 1.1	5.4 ± 5.1	5.7 ± 4.2	12 ± 12

Table S6. Summary of statistical analyses in Figure 6.

Outcomes	Test Used	Factors Tested	Test Statistic (df)	Main Effect p-values	Post Hoc Test	Significant Comparisons	Notes/Assumptions
Count	Two-way ANOVA	Patient, Condition	F(2,6) = 5.291 (Patient), F(4,8) = 0.9016 (Condition)	Patient: 0.0344, Condition: 0.5062	–	–	Assumptions met
Viability	Two-way ANOVA	Patient, Condition	F(2,6) = 1.764 (Patient), F(4,8) = 2.617 (Condition)	Patient: 0.2319, Condition: 0.1150	–	–	Assumptions met
MSCs	Friedman	Condition (Repeated)	$\chi^2(4) = 4.00$	p = 0.4752	–	–	Failed Spearman's (p = 0.0013), Failed D'Agostino–Pearson (p = 0.0492)
EPCs	Two-way ANOVA	Patient, Condition	F(2,6) = 13.73 (Patient), F(4,8) = 2.688 (Condition)	Patient: 0.0026, Condition: 0.109	–	–	Assumptions met
Pericytes	Two-way ANOVA	Patient, Condition	F(2,6) = 1.695 (Patient), F(4,8) = 5.926 (Condition)	Patient: 0.2433, Condition: 0.0162	Holm–Šidák	A vs. E (p = 0.0122)	Assumptions met
$CD34^{-}$ Pericytes	Two-way ANOVA	Patient, Condition	F(2,6) = 3.681 (Patient), F(4,8) = 11.97 (Condition)	Patient: 0.0736, Condition: 0.0019	Holm–Šidák	A vs. C (p = 0.0307), A vs. D (p = 0.0027), A vs. E (p = 0.0026), B vs. D (p = 0.0347), B vs. E (p = 0.0347)	Assumptions met
Macrophages	Two-way ANOVA	Patient, Condition	F(2,8) = 25.26 (Patient), F(4,8) = 0.9383 (Condition)	Patient: 0.0003, Condition: 0.4888	–	–	Assumptions met
$CD34^{dim}$	Two-way ANOVA	Patient, Condition	F(2,8) = 7.599 (Patient), F(4,8) = 1.203 (Condition)	Patient: 0.0141, Condition: 0.0363	Holm–Šidák	None Significant	Assumptions met
SA-ASC ($CD34^{dim}$)	Two-way ANOVA	Patient, Condition	F(2,8) = 6.652 (Patient), F(4,8) = 1.854 (Condition)	Patient: 0.0199, Condition: 0.2122	–	–	Assumptions met
TA Progenitors ($CD34^{dim}$)	Two-way ANOVA	Patient, Condition	F(2,8) = 2.700 (Patient), F(4,8) = 4.593 (Condition)	Patient: 0.0306, Condition: 0.5800	–	A vs. E (p = 0.0246)	Assumptions met

Comparison Key: MF [A], sEMD [B], iEMD (180 sec) [C], iEMD (270 sec) [D], iEMD (360 sec) [E]
